# Diagnostic delays in vasculitis and factors associated with time to diagnosis

**DOI:** 10.1186/s13023-021-01794-5

**Published:** 2021-04-21

**Authors:** Antoine G. Sreih, Keri Cronin, Dianne G. Shaw, Kalen Young, Cristina Burroughs, Joyce Kullman, Kirthi Machireddy, Carol A. McAlear, Peter A. Merkel, for the Vasculitis Patient-Powered Research Network

**Affiliations:** 1 Division of Rheumatology, University of Pennsylvania, White Building, 5th Floor, 3400 Spruce Street, Philadelphia, PA 19104 USA; 2 The Vasculitis Foundation, Kansas City, MO USA; 3 Department of Biostatistics, University of South Florida, Tampa, USA; 4 Vasculitis Patient-Powered Research Network, Philadelphia, PA USA

**Keywords:** Vasculitis, Diagnosis, Patient-reported data

## Abstract

**Background:**

Patients with vasculitis, a set of rare diseases, encounter delays in obtaining an accurate diagnosis which can lead to substantial morbidity and increased mortality. This study sought to describe the diagnostic journey of patients with vasculitis and identify factors associated with time to diagnosis.

**Methods:**

Patients with vasculitis enrolled in an online registry completed a two-stage study: Stage 1: survey of open-ended questions about patients’ diagnostic journeys and perceived factors associated with rapid or delayed diagnosis; Stage 2: survey with specific questions based on data from Stage 1 and additional investigator-identified factors.

**Results:**

375 patients with vasculitis participated in Stage 1; 456 patients participated in Stage 2. 85% of patients were seen by a healthcare provider within 3 months of the onset of symptoms. The median time to diagnosis of vasculitis was 7 months. 313/456 (73%) of patients were misdiagnosed initially. 40% of diagnoses were made in a hospital setting; 2% of diagnoses were made at a specialized vasculitis center. 60% of patients had at least 1 visit to an emergency room prior to diagnosis. Unemployment, time to travel to a medical center > 1 h, initial misdiagnosis, and delays in seeing a specialist were all associated with longer times to diagnosis. 373/456 (82%) of patients reported that a delayed diagnosis had negative consequences on their health.

**Conclusion:**

Patients with vasculitis encounter substantial delays in achieving an accurate diagnosis and these delays are associated with negative health consequences. Both patient-related factors and healthcare-related factors are associated with diagnostic delays.

## Background

Vasculitis is a heterogeneous group of rare diseases that affect blood vessels of different sizes and may result in organ failure or death. These disorders often have clinical presentations similar to other more common diseases and patients are often initially misdiagnosed and treated for other conditions prior to establishing the appropriate vasculitis diagnosis. Such delays in diagnosis can negatively impact clinical outcomes and frequently result in increased morbidity and mortality for all forms of vasculitis. Reducing delays in diagnosis can help alleviate this negative impact and improve clinical outcomes. Prior studies on diagnostic delays focused on single forms of vasculitis [1–4] or on patients seen at a tertiary care hospital [5] and were developed without patient engagement. This study aimed to gain a better understanding of the journey that patients with any form of vasculitis go through before receiving their diagnosis and identify factors associated with those delays. Patients were involved in the development, review, approval, and conduct of this study.

## Methods

The study had 3 aims: (1) identify the average amount of time that elapses between the onset of the first symptoms of vasculitis to the time of diagnosis; (2) identify factors associated with time to diagnosis of vasculitis; and (3) gain an understanding of patients’ perceptions of the consequences that result from delays in establishing a diagnosis of vasculitis. The study was conducted using a combination of qualitative and quantitative methods to understand patients’ experiences with working towards obtaining a diagnosis of vasculitis. The patients invited to join the study were members of the Vasculitis Patient-Powered Research Network (VPPRN, www.vpprn.org) with self-reported vasculitis. The VPPRN, founded in 2014, is an online registry for patients with all forms of vasculitis who are interested in participating in clinical research studies. The study was executed via a two-stage survey:

### Stage 1

This stage involved a short, open-ended qualitative survey asking patients to write in the factors they believed contributed to their diagnosis of vasculitis. The survey was administered to a series of randomly selected samples of patients until data saturation was reached. Data saturation was determined when a sample size was reached that was large enough for qualitative concept elicitation to allow for a richly textured understanding of the factors believed to contribute to a diagnosis of vasculitis and additional data would not meaningfully change the main results. Patients with diagnoses that had not been confirmed by a physician were excluded from analysis. Physician diagnosis of vasculitis was patient-reported. Incomplete responses were not included in the final analysis. The qualitative data from the Stage 1 survey responses was analyzed with NVivo software (QSR International Pty Ltd., Melbourne).

The data was examined using an inductive approach to coding the data by identifying distinct concepts and categories that emerged across several of the patients’ open-ended responses. This information enabled the breakdown of the responses into first-level concepts and second-level categories using a thematic synthesis approach. Responses were then reexamined to confirm that the categories accurately depicted the patient responses and to identify relationships among the concepts and categories. Nodes were then created to represent the emergent themes among concepts and categories. This information was then used to group the responses by speed of diagnosis and type of disease. The disease-specific coding involved calculating the frequency of each node for each of the different diseases.

### Stage 2

The results of the Stage 1 survey and input from health care providers were used to create a second survey. The Stage 2 survey was created using the factors that impacted the time to diagnosis mentioned by patients with the highest frequency. The second survey asked about the date of onset of symptoms and of diagnosis, the chronological order of symptoms experienced before the diagnosis was established, which tests were ordered and when they were completed, the type of providers seen by patients during their journey towards a diagnosis, and the type of provider and test that confirmed the diagnosis. Patients were also asked about other comorbid conditions. The Stage 2 survey is included in Supplementary Material.

Identified factors were classified as either intrinsic or extrinsic factors. Intrinsic factors are patient-related factors, such as the type of symptoms of vasculitis, demographics, socioeconomic status, and patients’ beliefs. Extrinsic factors are any factors related to healthcare professionals or health systems, such as access to healthcare and referral patterns. To gain a more complete understanding of patients’ access to care, additional questions were asked about specific social determinants of health, including health behaviors, social environment, income, and quality of health services.

This second survey was then reviewed and refined by a survey methodologist and pilot-tested by patient research partners on the VPPRN Research, Innovation, Planning, and Experiments (RIPE) and Recruitment, Education, and Communication (REC) working groups prior to being administered to VPPRN members through the online portal.

Two time periods prior to establishment of a diagnosis were assessed: (1) the *pre-encounter interval* is the period between the onset of the first symptoms of vasculitis and the initial encounter with a healthcare professional regarding those symptoms; (2) the *post-encounter interval* is the period between the first encounter with a healthcare provider and the establishment of the correct diagnosis. To minimize recall bias any patients diagnosed more than 5 years before the survey were not included in analysis. The median duration of diagnosis was calculated for all participants and for each sub-type of vasculitis. Discrete data generated by the survey is reported by counts; continuous data is reported by means $\pm$ standard deviation, and interquartile ranges (IQRs). Continuous variables were compared between groups using analysis of variance (ANOVA).

Univariate and multivariate logistic regression analysis was used to identify factors associated with delays in diagnosis. These factors included sex, age, race, geographic location, distance to closest major institution studying vasculitis, household income, marital status, presence of insurance, presence of co-morbidities, specialist consultation, time to specialist consultation, major testing to establish presence/absence of disease involved, time to obtain major testing, number of alternative diagnoses before vasculitis, and patient-related versus professional/health system-related factors. The Charlson Comorbidity Index was also used as a comorbidity summary measure [6]. All factors that had a statistically significant association with time to diagnosis in the univariate model were included in a multivariate model and analyzed using the forward selection method. All analyses were performed using STATA (StataCorp, College Station, Texas). A p value $\le$ 0.05 was considered to be statistically significant for both the univariate and multivariate model.

An Institutional Review Board reviewed and approved the study and all participants provided informed consent.

## Results

### Stage 1 survey results

Three-hundred and seventy-five patients responded to the initial Stage 1 survey. The majority of patients had a form of ANCA-associated vasculitis (eosinophilic granulomatosis with polyangiitis, granulomatosis with polyangiitis, or microscopic polyangiitis), but also included participants with Behçet’s disease, central nervous system vasculitis, cryoglobulinemic vasculitis, giant cell arteritis, IgA vasculitis, polyarteritis nodosa, Takayasu’s arteritis, and urticarial vasculitis.

The responses indicated that while some patients were promptly diagnosed, many patients experienced delays in diagnosis with a median time to diagnosis of 7 months.

The responses from the Stage 1 survey helped identify the types of variables that impact the amount of time it takes for a patient to be diagnosed. The results yielded variables that fell into the following categories: type of physician, presenting symptoms, patient disease experience, medical evaluation, patient resources, and medical intervention. The Stage 1 survey results indicated that patients felt that access to specialists and tertiary care had the greatest impact on the speed of their diagnosis. Participants who felt their physician was associated with their delayed diagnosis reported that the physician often lacked knowledge of vasculitis. Patients also attributed delays to their inability to access the appropriate specialist. Patients who felt they were quickly diagnosed with vasculitis often reported that they were able to easily access specialists who were willing to collaborate with other members of a care team, willing to refer the patient to other providers, and willing to run diagnostic tests.

The results of the Stage 1 survey were used to inform the design of the Stage 2 survey. The draft Stage 2 survey was pilot-tested by the VPPRN patient-partners and modified as needed before being administered electronically to patients through the VPPRN patient portal.

### Stage 2 survey results

**Fig. 1:**
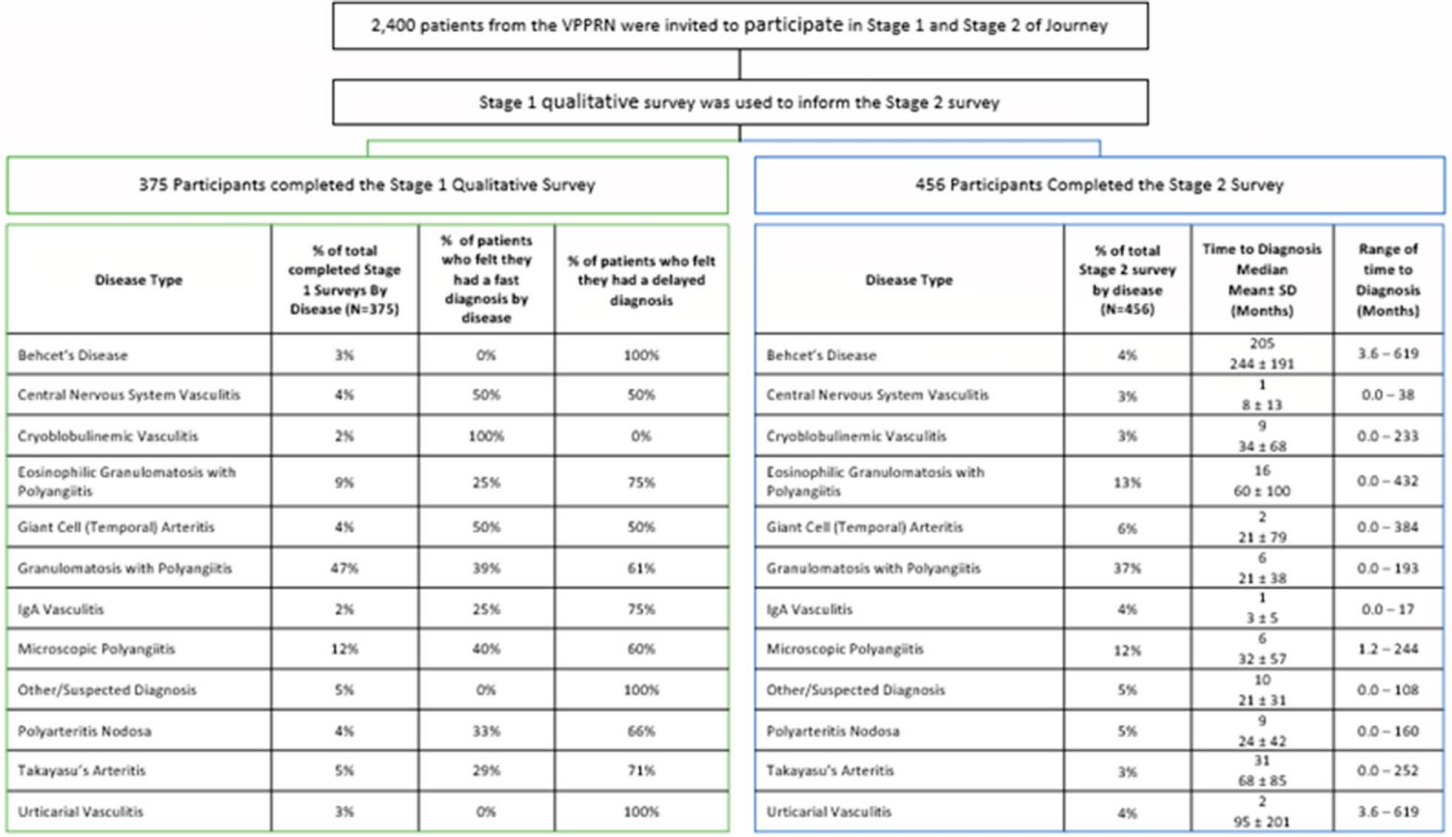
Participants in Stages 1 and 2 by Time to Diagnosis and Disease Type

**Fig. 2:**
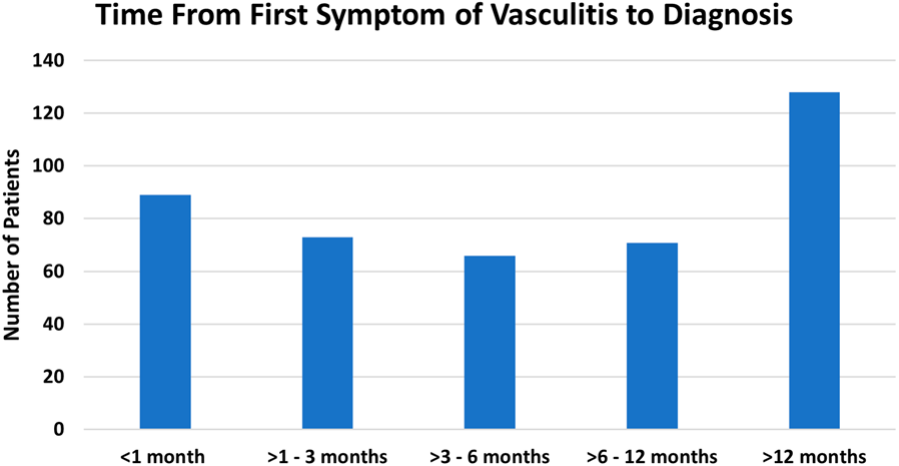
Time from first symptom of vasculitis to diagnosis

The Stage 2 survey was taken by 456 patients (Tables 1 and 2, Fig. 1). The mean age ± standard deviation was 56 ± 15 years, with 72% female, 95% Caucasian, 1% African American, 3% Asian, and 1% other race. Three percent of patients were Hispanic. Eighty percent of patients were from the United States. The median time to diagnosis of vasculitis was 7 months. Of the patients sampled, 50% were diagnosed within one year and 75% were diagnosed within two years. The time that elapsed prior to diagnosis varied for each of the different types of vasculitis. Patients with IgA-vasculitis experienced the shortest diagnosis time with a median of 1 month while patients with Behçet’s disease experienced the longest time to diagnosis with a median of 205 months (Fig. 2).

**Table 1:** Study subject demographics by disease

Disease type	Number of patients	Mean age ± SD (median)	Female N (%)	Caucasian N (%)
Behçet’s disease	17	45 ± 13 (41)	15 (88%)	15 (94%)
Central nervous system vasculitis	15	53 ± 11 (55)	12 (80%)	13 (87%)
Cryoglobulinemic vasculitis	15	63 ± 14 (67)	13 (87%)	14 (93%)
Eosinophilic granulomatosis with polyangiitis	58	59 ± 12 (60)	40 (69%)	54 (93%)
Giant cell arteritis	26	70 ± 9 (73)	20 (77%)	24 (92%)
Granulomatosis with polyangiitis	169	56 ± 15 (59)	110 (65%)	164 (98%)
IgA-vasculitis	18	49 ± 19 (53)	12 (67%)	17 (94%)
Microscopic polyangiitis	53	57 ± 14 (61)	43 (81%)	52 (98%)
Polyarteritis nodosa	21	47 ± 20 (50)	11 (52%)	19 (90%)
Takayasu’s arteritis	21	43 ± 18 (38)	20 (95%)	19 (90%)
Urticarial vasculitis	15	57 ± 12 (60)	12 (80%)	15 (100%)
Other	28	54 ± 14 (58)	22 (79%)	26 (93%)
Total for all types of vasculitis	456	56 ± 15 (59)	330 (72%)	432 (95%)

**Table 2:** Study subject demographics for entire study population

Demographics	N = 456 (%)
Sex	
Female	330 (72%)
Male	126 (28%)
Race	
American Indian or Alaskan Native	7 (2%)
Asian	13 (3%)
Black or African American	6 (1%)
Native Hawaiian or Pacific Islander	1 (0%)
White	390 (95%)
Multiple races	1 (0%)
Decline to answer	0
Ethnicity	
Hispanic, Latino, or of Spanish Origin	12 (3%)
Not Hispanic, Latino, or of Spanish Origin	418 (92%)
Do not know	16 (4%)
Missing	2 (0%)
Decline to answer	8 (2)
Location	
USA or Canada	370 (81%)
Outside USA or Canada	86 (19%)

Level of education	N = 456 (%)
Grade 8 or less	19 (4%)
Some high school	21 (5%)
High school graduate or GED	35 (8%)
Some college. No bachelor's degree	142 (31%)
Bachelor's degree	111 (24%)
Post-bachelor's degree awarded	118 (26%)
Decline to answer	8 (2%)
Employment status	
Disabled (unable to work)	16 (4%)
Student (not working)	45 (10%)
Employed with income	294 (64%)
Employed without income (volunteer)	3 (1%)
Homemaker	17 (4%)
Retired	64 (14%)
Decline to answer	5 (1%)
Household income (US Dollars)	
Less than $9999	24 (5%)
$10,000 to $29,000	43 (9%)
$30,000 to $39,000	50 (11%)
$50,000 to $99,000	126 (28%)

The majority of patients with vasculitis (73%) were initially misdiagnosed (Table 3). The most common misdiagnoses were infection (33%) and autoimmune disease (29%). Patient received a median of 5 misdiagnoses before obtaining a correct diagnosis of vasculitis.

**Table 3:** Initial diagnosis given before the diagnosis of vasculitis was established

Initial diagnosis	
Infection	33%
Allergies	21%
Autoimmune disease	19%
Not a “real” illness	11%
Fibromyalgia syndrome	6%
Cancer	5%
Other	49%

An initial misdiagnosis was associated with substantial delays in achieving the correct diagnosis of vasculitis. It took a mean of 3.9 ± 8 years for patients who were initially misdiagnosed to obtain their diagnosis of vasculitis versus a mean of 1.58 ± 5 years to get a diagnosis of vasculitis for patients who were not initially misdiagnosed.

The symptoms that patients presented with impacted the amount of time that elapsed prior to reaching a diagnosis. Patients with genital ulcers (mean 14.86 ± 16 years), blood in their stool (mean 9.69 ± 17 years), scalp tenderness (mean 8.94 ± 15 years), mouth sores (mean 8.26 ± 13 years), and nausea or digestive issues (mean 6.49 ± 12 years) experienced longer delays in diagnosis than other patients (mean 3.3 ± 7 years). In contrast, patients with bleeding in their lungs (mean 1.53 ± 3 years) achieved a diagnoses more rapidly than other patients (mean 3.3 ± 7 years). The techniques used to diagnosis the presenting symptoms also had an impact on the time it took to diagnosis the patient’s condition. Patients who had biopsies (mean 2.84 ± 7 years), or angiography (mean 2.82 ± 6 years), or other radiological testing (mean 2.78 ± 6 years), were diagnosed faster than patients who did not receive these forms of diagnostic testing (3.3 ± 7 years).

Prior to achieving a diagnosis of vasculitis 60% (238, N = 400) had at least 1 visit to an emergency room (ER) prior to obtaining a diagnosis of vasculitis. Of these patients, 5% visited the ER more than 10 times before they were finally diagnosed. Of the 40% of patients who were diagnosed in a hospital, only 5% were diagnosed in the ER.

The vast majority of patients (91.6%) were diagnosed by medical specialists. Patients who were initially referred to specialists (mean 2.67 ± 7 years) were diagnosed faster than patients who initially were not referred by specialists (mean 4.11 ± 8 years).

In a multivariate analysis, factors that were associated with longer delays in diagnosis of vasculitis included being unemployed, having a medical center located farther than 1 h away, receiving an initial misdiagnosis, and experiencing delays in being able to see a specialist (Table 4).

**Table 4:** Factors associated with time to diagnosis of vasculitis

Factors	Coefficient (95% CI)	p value
*Patient-related factors*		
Time to travel to healthcare site > 1 h	2.6 (0.6–4.5)	< 0.01
Patient location (North America)	1.2 (− 2.0–3.8)	0.76
Single or divorced or widowed	1.1 (− 1.0–3.2)	0.29
Household income > $50,000/year	− 1.5 (− 4.2–0.6)	0.18
Female	− 1.5 (− 4.0–0.5)	0.15
Caucasian race	− 1.5 (− 6.0–3.0)	0.54
Charlson score > 1	− 1.5 (− 3.9–0.4)	0.12
Employed	− 2.4 (− 4.0–− 0.4)	0.02
*Healthcare-related factors*		
Time to see a specialist > 1 month	2.4 (0.3–4.6)	0.03
Misdiagnosis	2.3 (0.1–4.5)	0.03
Laboratory studies ordered initially	0.2 (− 1.6–2.0)	0.80
Referral delays due to insurance	− 0.3 (− 2.5–2.5)	0.98
Specialist involved initially	− 1.3 (− 3.1–0.6)	0.18

The table reports on the results of a multivariate analysis. A positive coefficient indicates a longer time to diagnosis and a negative one indicates a shorter time to diagnosis

CI: confidence interval; $: US dollars

Of the patients who experienced a delayed diagnosis delays, 82% reported that the delay had a negative impact on their health. These individuals often reported that the delay led to their condition worsening (55%), to them losing their job (16%), and to them becoming disabled (11%).

## Discussion

Gaining a better understanding of the factors that contribute to delays in the diagnosis of patients with vasculitis creates a more detailed picture of the presentation of this group of diseases. Subsequent mitigation of some of these factors could lead to earlier diagnosis, initiation of appropriate treatment, and ultimately minimizing the negative impact of these organ- and life-threatening diseases.

The present study assessed multiple clinical and health system factors that patients and clinicians identified as potentially leading to delays in obtaining a diagnosis of vasculitis. Factors found significantly associated with a delay in diagnosis included a delay in the patient being seen by a specialist, unemployment, and travel time to a healthcare site. Not surprisingly, an initial misdiagnosis of a disease other than vasculitis also led to delays in diagnosis and such initial misdiagnoses remain quite common. The results from the present study substantively extend prior similar work in vasculitis by studying multiple forms of vasculitis, asking patients about their experiences directly, and engaging patients in the project to help ensure that patients’ perspectives on the problem of delays in diagnosis influenced the design of the study [1–4]. The current study also aligns with and extends findings from studies of arriving at diagnoses for other systemic rheumatic diseases and cancer [7–13].

Strengths of this study include the relatively large number of patients from which data were collected and the inclusion of patients’ perspectives in the design of the project and the surveys.

There are also limitations to the current study to consider. The study population was derived from the online VPPRN which has a disproportionate number of patients who are female, Caucasian, from North America, and with ANCA-associated vasculitis, thus reducing the full generalizability of the findings. The reliability of patient-reported diagnoses of vasculitis is also a potential limitation. However, a recent study in the VPPRN found that patient self-reported diagnosis of ANCA-associated vasculitis to be reliable, with 86–96% of patients fulfilling the 1990 American College of Rheumatology classification criteria and/or the 2012 Chapel Hill Consensus Conference definitions [14]. Another limitation is selection bias as it may be assumed that patients with a delay in diagnosis would be more likely to participate in a study investigating delays in diagnosis. To minimize this possible effect, the study sought to describe the diagnostic journey of patients with vasculitis from the onset of symptoms to diagnosis and identify factors associated with the time to diagnosis, not just delays. All study recruitment materials and study descriptions emphasized the journey to diagnosis whether fast or delayed. The Stage 1 data reported by disease illustrates that the perceived fast or delayed diagnosis varied greatly. The Stage 2 survey data illustrates a similar time to diagnosis when broken down by disease, see Fig. 1. The percentage of patients who participated in the Stage 1 qualitative study were proportional to those who participated in Stage 2.

## Conclusion

The results of this study illustrate that various factors, both patient-related and healthcare system-related, lead to delays in diagnosis for patients with vasculitis and that these delays can often have a negative impact on patient health and quality of life. Thus, creating ways to accelerate arriving at the correct diagnosis for patients with vasculitis is of the utmost importance. These conclusions indicate that healthcare providers should be better educated on the various ways in which vasculitis can present clinically and what approaches to diagnosis are appropriate in cases of potential vasculitis. Increasing awareness and understanding of vasculitis disorders will hopefully help to streamline the referral process and help eliminate delays in diagnosis.

## Data Availability

All data generated during and/or analyzed during the current study are not publicly available because the data includes participants’ personal health information (e.g. diagnosis, age) but are available from the corresponding author on reasonable request.

## Abbreviations

VPPRN: Vasculitis Patient-Powered Research Network
